# Interleukin 17A Participates in Renal Inflammation Associated to Experimental and Human Hypertension

**DOI:** 10.3389/fphar.2019.01015

**Published:** 2019-09-13

**Authors:** Macarena Orejudo, Raul R. Rodrigues-Diez, Raquel Rodrigues-Diez, Ana Garcia-Redondo, Laura Santos-Sánchez, Javier Rández-Garbayo, Pablo Cannata-Ortiz, Adrian M. Ramos, Alberto Ortiz, Rafael Selgas, Sergio Mezzano, Carolina Lavoz, Marta Ruiz-Ortega

**Affiliations:** ^1^Cellular Biology in Renal Diseases Laboratory, IIS-Fundación Jiménez Díaz, Universidad Autónoma de Madrid, Madrid, Spain; ^2^Red de Investigación Renal (REDinREN), Instituto de Salud Carlos III (ISCIII), Madrid, Spain; ^3^Pharmacology Department, Facultad de Medicina, Universidad Autónoma de Madrid, Madrid, Spain; ^4^Division of Nephrology and Hypertension, IIS-Fundación Jiménez Díaz-Universidad Autónoma de Madrid, Madrid, Spain; ^5^Division of Nephrology and Hypertension, IIS-Fundación Jiménez Díaz-Universidad Autónoma de Madrid, Madrid, Spain; ^6^Laboratory of Nephrology, Fundación de Investigación Biomédica Hospital Universitario la Paz (FIBHULP- IdiPAZ), Universidad Autónoma de Madrid, Madrid, Spain; ^7^Division of Nephrology, School of Medicine, Universidad Austral, Valdivia, Chile

**Keywords:** IL-17A, hypertension, renal pathology, IL-17A neutralization, inflammation

## Abstract

Hypertension is now considered as an inflammatory disease, and the kidney is a key end-organ target. Experimental and clinical studies suggest that interleukin 17A (IL-17A) is a promising therapeutic target in immune and chronic inflammatory diseases, including hypertension and kidney disease. Elevated circulating IL-17A levels have been observed in hypertensive patients. Our aim was to investigate whether chronically elevated circulating IL-17A levels could contribute to kidney damage, using a murine model of systemic IL-17A administration. Blood pressure increased after 14 days of IL-17A infusion in mice when compared with that in control mice, and this was associated to kidney infiltration by inflammatory cells, including CD3^+^ and CD4^+^ lymphocytes and neutrophils. Moreover, proinflammatory factors and inflammatory-related intracellular mechanisms were upregulated in kidneys from IL-17A-infused mice. In line with these findings, in the model of angiotensin II infusion in mice, IL-17A blockade, using an anti-IL17A neutralizing antibody, reduced kidney inflammatory cell infiltrates and chemokine overexpression. In kidney biopsies from patients with hypertensive nephrosclerosis, IL-17A positive cells, mainly Th17 and γδ T lymphocytes, were found. Overall, the results support a pathogenic role of IL-17A in hypertensive kidney disease-associated inflammation. Therapeutic approaches targeting this cytokine should be explored to prevent hypertension-induced kidney injury.

## Introduction

Hypertension is a prevalent disorder and the second leading cause of kidney failure after diabetes (Coffman, 2011). It may be complicated by target organ damage, including cardiovascular, brain, and kidney injuries. Despite the availability of antihypertensive drugs, blood pressure control is frequently suboptimal, and end-organ damage is still common. Thus, improved organ protection strategies are needed. The etiology of essential hypertension remains unclear and is still a matter of intense debate. In the 1960s, experimental work showed that inflammation was involved in hypertension (White and Grollman, 1964). Later on, several independent clinical studies have described elevated levels of circulating proinflammatory cytokines in hypertensive patients, and extensive preclinical research has unraveled a role for innate, cellular, and humoral immunity in the pathogenesis of hypertension and its complications (McMaster et al., 2015). Interleukin 17A (IL-17A), the effector cytokine of Th17 cells, has emerged as a promising therapeutic target in immune and chronic inflammatory diseases, including hypertension and chronic kidney disease (Caillon and Schiffrin, 2016; Solak et al., 2016; Cortvrindt et al., 2017). Initial studies described the involvement of IL-17A in pathogen clearance during infection (Fossiez et al., 1998). Now, IL-17A is considered as a pleiotropic cytokine involved in tissue inflammation and destruction through the increased expression of pro-inflammatory cytokines, chemokines, adhesion molecules, and matrix metalloproteases (Von Vietinghoff and Ley, 2010; Cortvrindt et al., 2017).

Regarding hypertension, elevated circulating IL-17A levels have been described in hypertensive patients and in patients with hypertension-associated diseases, including systemic lupus erythematosus, preeclampsia, and chronic allograft rejection (McMaster et al., 2015; Yao et al., 2015; Beringer et al., 2016: Loverre et al., 2011; Chehimi et al., 2017). Experimental data support a role for Th17 cells and its effector cytokine IL-17A in the pathogenesis of hypertension (McMaster et al., 2015). A pioneer study showed that mice lacking T and B cells did not develop hypertension in response to angiotensin II (AngII) infusion. Moreover, adoptive transfer of T, but not B, cells restored the hypertensive response (Kvakan et al., 2009). Experimental studies in genetically modified mice for IL17A or its receptor and studies using neutralizing antibodies against IL-17A further support a role for this cytokine in the pathogenesis of hypertension and associated renal and vascular end-organ dysfunction (Madhur et al., 2010; Nguyen et al., 2013; Saleh et al., 2016; Guzik and Touyz, 2017). Even though IL-17A has emerged as a key regulator of the immune response in several disease conditions, its regulation is still poorly understood. Interestingly, preclinical studies have reported both pro- and anti-atherogenic roles of IL-17A (Gong et al., 2015), showing that the function of IL-17A in disease conditions may be more complex than previously thought. Importantly, IL-17A blockers have proven effective to treat ankylosing spondylitis, chronic plaque psoriasis, and psoriatic arthritis and are being studied in other inflammatory diseases (Leonardi et al., 2012; Baeten et al., 2013; Baeten et al., 2015; Mease et al., 2015). Interestingly, administration of recombinant IL-17A results in increased systolic blood pressure in mice, and this was associated to endothelial dysfunction and vascular damage (Nguyen et al., 2013). However, the kidney effects of IL-17A in the context of hypertension remain to be investigated.

In this paper, we tested the hypothesis that chronically elevated circulating IL-17A levels could contribute to renal damage and the potential mechanisms involved. To this aim, we have developed a novel model of continuous infusion of systemic IL-17A in mice, resembling chronically elevated IL-17A levels found in prehypertensive patients (Yao et al., 2015).

## Materials and Methods

### Experimental Models

All procedures on animals were performed according to recommendations by the European Community, and the protocol was approved by Instituto de Investigación Sanitaria Fundación Jiménez Díaz Animal Research Ethical Committee and by Comunidad de Madrid.

To evaluate the renal effects of chronic exposure to elevated circulating IL-17A levels, an experimental model of continuous infusion of systemic IL-17A was developed in adult male C57BL/6 mice (9–12 weeks old, 20 g). Recombinant IL-17A administration was done using subcutaneous osmotic minipumps (Model 2002; Alza Corp., Palo Alto, CA, USA), at the dose of 1.5 ng/g of body weight, and mice were studied after 14 days. This dose was based on previous data observed in preclinical mice studies and serum IL-17A levels observed in prehypertensive patients (Madhur et al., 2010; Nguyen et al., 2013; Yao et al., 2015).

Systemic infusion of AngII to adult male C57BL/6 mice was done using osmotic minipumps (at the dose of 1,000 ng/kg/min for 14 days), as described (Esteban et al., 2003; Kvakan et al., 2009; Madhur et al., 2010; Alique et al., 2014). One group of mice was also treated with a neutralizing anti-IL-17A antibody (100 µg/mouse i.p. every 4 days), at a dose previously described (Rodrigues-Díez et al., 2014). This dose of AngII was previously shown to cause after 3 days an early kidney inflammatory cell infiltration (Esteban et al., 2003) associated to upregulation of renal *Ngal* gene expression and elevated urinary neutrophil gelatinase-associated lipocalin (NGAL) levels (Suarez-Alvarez et al., 2017). After 2 weeks of AngII infusion, persistent kidney inflammation was observed, but there was no decrease in renal function (as assessed by serum creatinine), whereas there was no fibrosis (Alique et al., 2014). By 4 weeks, kidney fibrosis and proteinuria were evident, but no significant changes in serum creatinine levels were found (Lu et al., 2019).

In mice experiments, control animals were untreated or infused with saline adult male C57BL/6 mice, showing no differences between those groups. Therefore, all experiments were compared with untreated mice (considered as controls in the text).

Hypertension-induced renal damage was also evaluated in renal biopsies of male Wistar rats continuously infused with 100 ng/kg/min AngII for 14 days (subcutaneous osmotic minipumps; Model 2002). This model is characterized by increased blood pressure and kidney inflammatory cell infiltration and fibrosis, as previously described (Ruiz-Ortega et al., 2001; Lavoz et al., 2012; Wang et al., 2015). In addition, 16-week-old spontaneously hypertensive (SHR) rats were also studied. SHR rats presented elevated blood pressure, albuminuria, and renal fibrosis, compared with control WKY of the same age, as described (Lavoz et al., 2012).

### Sample Processing

Spot urine samples were collected once a week from all mice and analyzed for albumin by enzyme-linked immunosorbent assay (ELISA) (ALPCO Immunoassasys, Salem, NH, USA). Blood samples were obtained by cardiac puncture at the time of sacrifice, and blood was centrifuged at 3,000 rpm for 10 min to obtain serum that was stored at -80°C until analysis (standard biochemical determinations: blood urea nitrogen and creatinine), as previously described (Martin-Sanchez et al., 2018).

At the time of sacrifice, animals were anesthetized with 5 mg/kg xylazine (Rompun, Bayer AG) and 35 mg/kg ketamine (Ketolar, Pfizer), and the kidneys were perfused *in situ* with cold saline before removal. A piece of the kidney (2/3) was fixed, embedded in paraffin, and used for immunohistochemistry, and the rest was snap-frozen in liquid nitrogen for renal cortex RNA and protein studies.

### Systolic Blood Pressure Measurements

The LE5001 noninvasive blood pressure acquisition system (Panlab, Hardvard apparatus) and the appropriated cuff and transducer (76-0432 for mice; Panlab Hardvard Apparatus) were used. The blood pressure measurements were done in a quiet and temperature-regulated area (+/-22°C). Animals where preheated (37°C, 10 min) before measurements and maintained at 35°C. The occlusion cuff was placed at the base of the tail, and the transducer was placed adjacent to the occlusion cuff. In each session, 10 to 15 measurements per animal were done, and the first 5 data were excluded. Mice were habituated for at least 3 days before experiments. Systolic blood pressure is expressed as the mean of 5 to 10 measurements each day.

### Clinical Data and Human Renal Biopsies

Percutaneous renal biopsies performed at the Division of Nephrology, Austral University, Valdivia, Chile were studied if samples were available after completing the diagnostic workup and if patients signed written inform consent forms approved by local hospital ethics committee (Comité de Ética de Investigación, Servicio de Salud Valdivia, Ministerio de Salud, Chile). The study is adhered to the Declaration of Helsinki. All patients (n = 20, age 56.7 ± 17.1 years; male/female ratio: 7/12) had hypertension, and the indication of renal biopsy was the diagnostic workup of an abnormal urinalysis (mainly the presence of proteinuria) and/or decreased renal function. Thus, mean proteinuria values were 200 ± 130 mg/dl and serum creatinine 2.0 mg ± 1.3 mg/dl. The key inclusion criterion was a histopathological diagnosis of nephroangiosclerosis that was attributed to hypertension in the absence of evidence of other separate kidney diseases, defined by the findings at light microscopy hematoxylin–eosin (H&E), Masson trichrome and periodic acid-Schiff (PAS) staining, with negative immunofluorescence for immune deposits and absence of electron immune deposits at electron microscopy. This diagnosis represented 2% of renal biopsies. A limitation of the present study is the absence of control kidneys (from healthy humans) that were not possible to obtain.

### Immunohistochemistry and Immunofluorescence

Paraffin-embedded 3-μm (immunostaining, H&E, PAS) or 5-μm (picrosirius red) thick kidney sections were stained using standard histology procedures: H&E (for structural evaluation), PAS (to evaluate renal lesions), and picrosirius red (to evaluate renal fibrosis). Samples were mounted in nonaqueous dibutylphthalate polystyrene xylene medium.

Histopathological analysis was performed by PAS-stained paraffin-embedded murine kidney samples in a blinded fashion by a pathologist examined in a Nikon Eclipse E400 microscope. Kidney damage was evaluated by a semiquantitative score: grade 0, normal; grade 1, segmental lesion <25%; grade 2, 25–50%; grade 3, 50–75%; grade 4, 75–100%, as described (Shen et al., 2018). PAS evaluation was realized by visual approach, using parameters such as mesangial proliferation (glomeruli parameter); regeneration, tubular atrophy, and tubular dilatation (tubular parameters); fibrosis, peritubular, and perivascular inflammatory infiltrate. Total renal damage is the sum of those parameters. At least 20 glomeruli were analyzed in each group. Sirius Red staining was used to evaluate tubulointerstitial fibrosis index. The areas of interstitial fibrosis were evaluated in 10 random 400× magnification fields stained. For fibrosis quantification, the percentage stained area out of the total area was calculated in five randomly chosen fields (×200 magnification) using Image-Pro Plus software (Media Cybernetics Inc. Rockville, MD, USA), and results were expressed as fold-change over control. Picrosirius red sections were also viewed with polarization contrast illumination in a Leica DMD108 microscope (Leica Microsystems, Wetzlar, Germany).

Immunohistochemistry was performed in a Dako Autostainer (Dako), as described previously (Lavoz et al., 2018). Firstly, endogenous peroxidase was blocked, and then, sections were incubated for 30 min at room temperature or overnight at 4ºC with primary antibodies as described later, followed by 1 h of secondary antibody incubation (AP132B and AP124B, dilution: 1:200; Merk Millipore). Finally, the EnVision™ DuoFLEX Doublestain System using 3,3’-diaminobenzidine was used. Sections were counterstained with hematoxylin and evaluated by optical microscopy. Images were obtained with a Nikon Eclipse E400 microscope and analyzed with Image Pro-plus (Media Cybernetics Inc. Rockville, MD, USA). Negative controls included nonspecific immunoglobulin and no primary antibody (not shown). IL-17A and interstitial infiltrating cells were detected by staining with anti-IL-17A (ab9565, dilution: 1:100, Abcam, Cambridge, U.K.), anti-F4/80 for monocytes/macrophages (MCA497, dilution: 1:70, Bio-Rad), anti-CD3 for T lymphocytes (A0452, dilution: 1:150, Dako), anti-CD4 for T helper lymphocytes (IS649, ready to use, Dako), anti-γδ-TCR for γδ lymphocytes (331201, dilution: 1:250; Biolegend, San Diego, CA, USA), anti-myeloperoxidase for neutrophils (A0398, dilution: 1:1,000, Dako), or anti-tryptase for mast cells (IR640; ready to use, Dako) antibodies.

For immunofluorescence staining, primary antibodies were followed by their corresponding anti-IgG Alexa488- or Alexa633-conjugated secondary antibody. Nuclei were counterstained with 4,6-diamidino-2-phenylindole. Samples were mounted in Prolong Gold antifade reagent (Invitrogen, Life Technologies Corporation) and examined by using a Leica DM-IRB confocal microscope.

### Protein Studies

Proteins were obtained from mouse or rat kidneys using lysis buffer (50 mmol/l Tris-hydrochloric acid, 150 mol/l sodium chloride, 2 mmol/l ethylenediaminetetraacetic acid, 2 mmol/l egtazic acid, 0.2% Triton X-100, 0.3% IGEPAL, 10 μl/ml proteinase inhibitor cocktail, 0.2 mmol/l phenylmethylsulfonyl fluoride, and 0.2 mmol/l orthovanadate). Protein levels were quantified using a Pierce^™^ BCA protein assay kit (Thermo Scientific, Rockford, IL, USA). For Western blotting, cell protein extracts (20–25 μg/lane) or cell supernatants (25–30 μl/lane) were separated on 6–12% polyacrylamide-sodium dodecyl sulfate gels under reducing conditions. Samples were then transferred onto polyvinylidene difluoride membranes (BioRad, Spain), blocked with Tris-buffered saline/5% nonfat milk/0.05% Tween-20, and incubated overnight at 4ºC with the corresponding primary antibodies. The quality of proteins and efficacy of protein transfer were evaluated by Red Ponceau staining (data not shown). After washing, membranes were incubated with the appropriate horseradish peroxidase-conjugated secondary antibody (Amersham Biosciences) and developed using Luminata^™^ Crescendo Western horseradish peroxidase substrate (Millipore). Digital chemiluminescence images were taken by LAS 4000 (GEHealthcare) and quantified by Quantity One^®^ software. The following primary antibodies were employed [dilution]: p-AKT1/2/3 (sc-271966 [1:500], Santa Cruz Biotechnology), pSmad2/3 (sc-11760 [1:500] Santa Cruz Biotechnology), kallikrein (420308 antibody, 1:100 dilution, Calbiochem), glyceraldehyde 3-phosphate dehydrogenase ([1:5,000]; Chemicon International), and α-tubulin (T5168 [1:10,000], Sigma-Aldrich).

### Gene Expression Studies

RNA was isolated with the TriPure reagent (Roche) from renal tissue pulverized in a metallic chamber. Complementary DNA was synthesized by a High Capacity cDNA Archive kit (Applied Biosystems) using 2 μg of total RNA primed with random hexamer primers following the manufacturer’s instructions. Next, quantitative gene expression analysis was performed by real-time PCR on an AB7500 fast real-time PCR system (Applied Biosystems) using fluorogenic TaqMan MGB probes and primers designed by Assay-on-Demand™ gene expression products. Mouse assay IDs were: *Kidney injury molecule 1 (Kim1 or Havcr1)*, Mm00506686_m1; *Neutrophil gelatinase-associated lipocalin (Ngal or Lcn2)*, Mm01324470_m1; *Fibronectin* (Fn1), Mm01256744_m1; *type I collagen* (Col1a2), Mm00483888_m1; *Plasminogen activator inhibitor* (*pai-1* or Serpine), Mm00435858_m1; *Monocyte chemoatractant protein1 Mcp1* (Ccl2), Mm00441242_m1; *Rantes* (Ccl5), Mm01302428_m1; *IL23a*, Mm00518984_m1; *IL23r*, Mm00519943_m1, *Klk-1*, Mm00834006_g1, *IL6*, Mm00446190_m1; *Icam1*, Mm00516023_m1; *Vcam1*, Mm01320970_m1; *Tnf*α, Mm00443258_m1; *IL1β*, Mm00434228_m1; *Tlr4*, Mm01302428_m1. Data were normalized to mouse *gapdh*: Mm99999915_g1. The messenger RNA (mRNA) copy numbers were calculated for each sample by the instrument software using the Ct value (“arithmetic fit point analysis for the lightcycler”). Results were expressed in copy numbers, calculated relative to unstimulated cells after normalization against glyceraldehyde 3-phosphate dehydrogenase.

### Statistical Analysis

Results throughout the text are expressed as mean ± SEM of fold increase over control. Differences between groups were assessed by Student t (cells), Mann–Whitney (mice) and nonparametric analysis of variance (rats) tests. Statistical significance was assumed when a null hypothesis could be rejected at p < 0.05. Statistical analysis was performed using the GraphPad Prism software (GrahPad Software, San Diego California USA).

## Results

### Systemic Infusion of Interleukin 17A in Mice Increased Blood Pressure and Induced Kidney Inflammation

**Figure 1:**
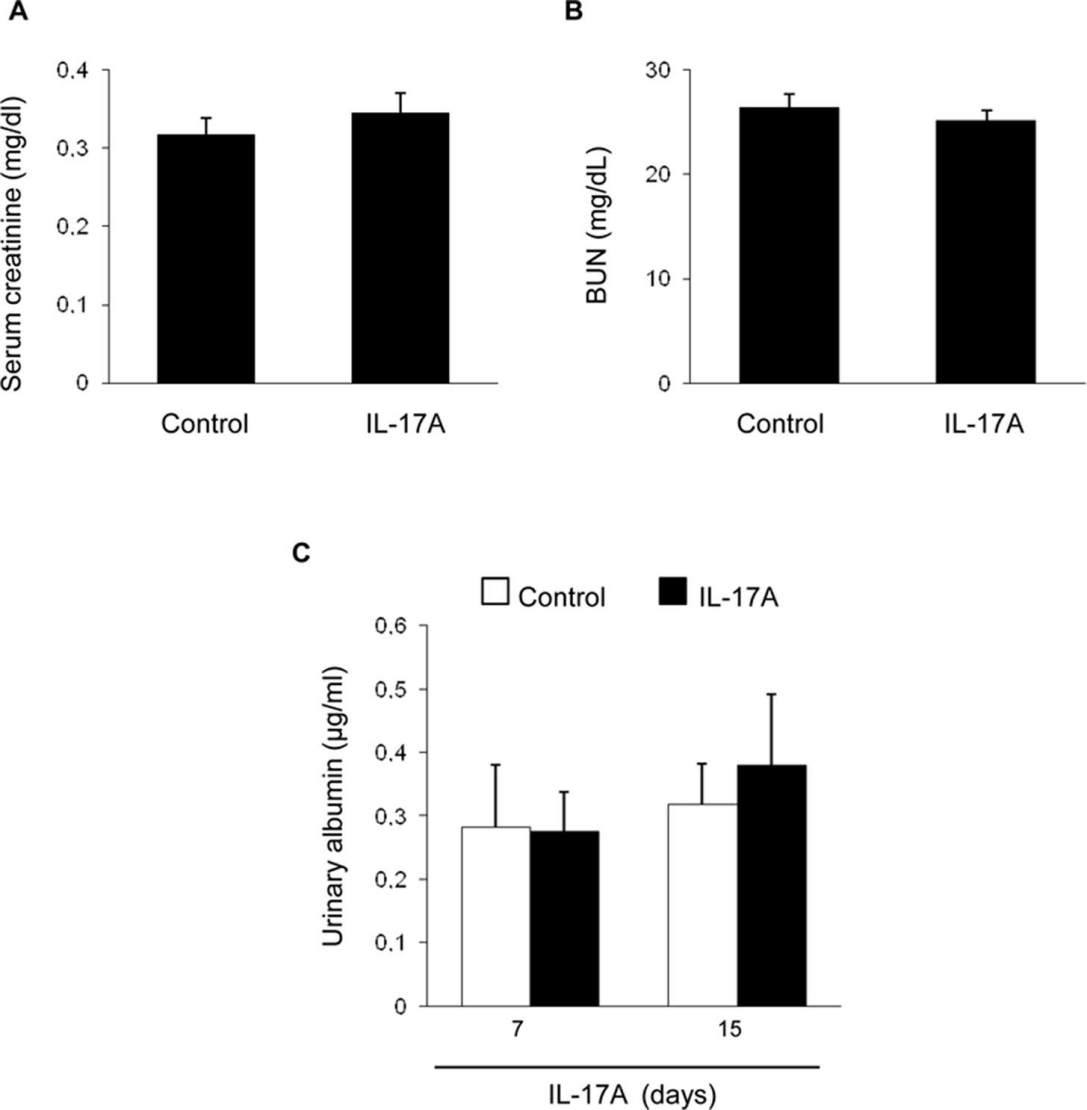
Kidney function parameters in mice with continuous systemic IL-17A infusion. IL-17A was continuously infused in mice by subcutaneous osmotic minipumps, at a dose of 1.5 ng/g for 14 days. Serum creatinine **(A)** and blood urea nitrogen **(B)** levels were assessed at 14 days. **(C)** Albuminuria was evaluated at 7 and 14 days. Data are expressed as mean ± S.E.M. of n = 6–8 mice per group. p = n.s. vs. control.

**Figure 2:**
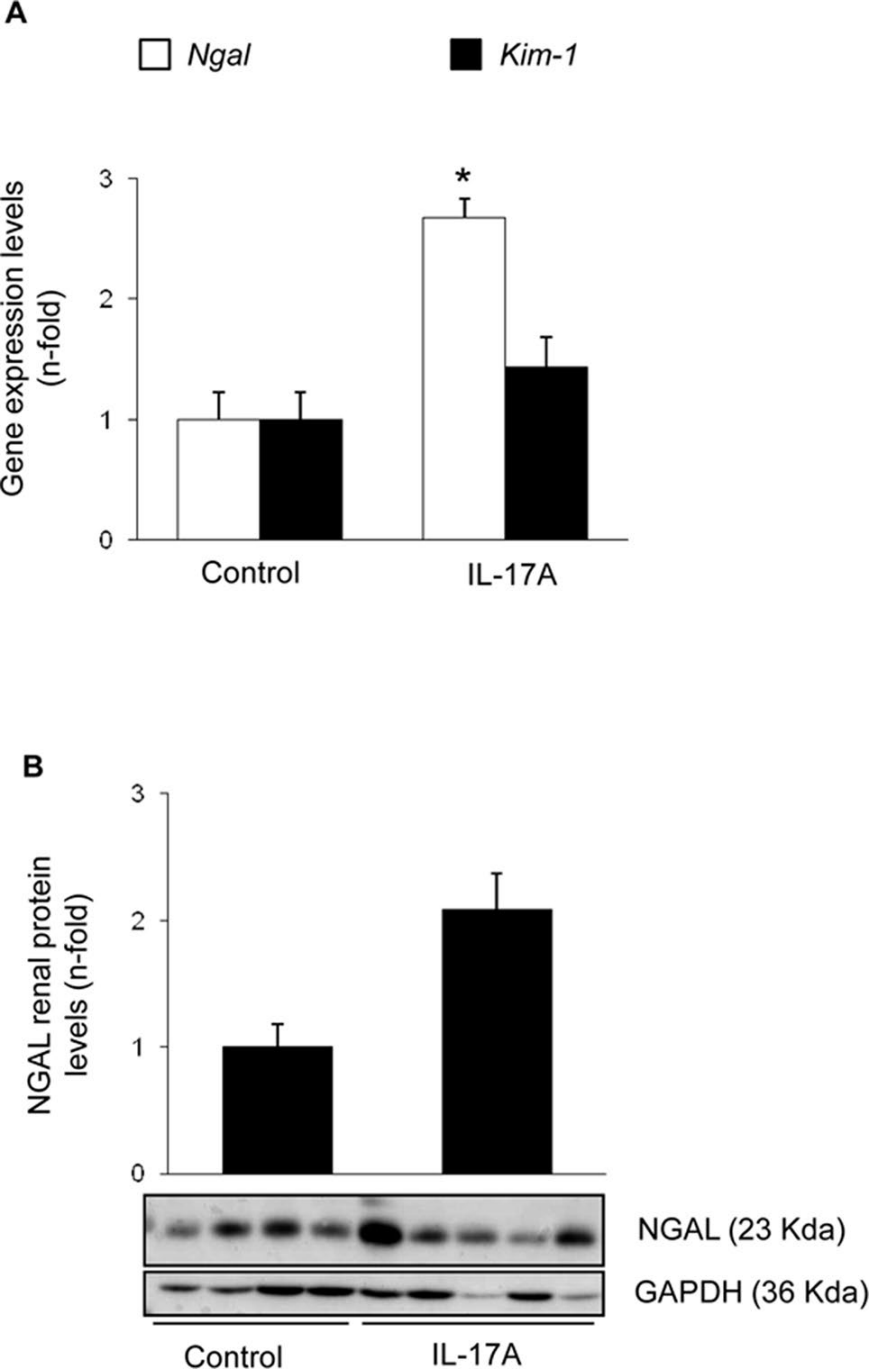
Kidney injury parameters in mice with continuous systemic IL-17A infusion. **(A)** RNA was obtained from total kidney extracts, and gene expression of the biomarkers of kidney injury *Ngal* and *Kim-1* were studied by real-time PCR. **(B)** Total proteins were isolated from whole mouse kidneys, and NGAL levels were evaluated by Western blot. Figure shows data as the mean ± S.E.M. of 6–8 mice per group. *p < 0.05 vs. control.

Systemic IL-17A infusion in mice increased systolic blood pressure over control values (117 ± 4 mm Hg vs. 88 ± 3 mm Hg; p < 0.05 vs control, n = 6–8 mice per group at 14 days). However, IL-17A infusion did not modify renal function as assessed by serum urea and creatinine (**Figures 1A, B**) or spot albuminuria (**Figure 1C**). These data suggest no significant impact of IL-17A infusion on parameters of kidney function or injury that are commonly used in the clinic, at the time points studied. A more sensitive assessment of kidney injury explored kidney gene expression of two biomarkers, *Kim-1* and *Ngal*. There was a nonsignificant trend toward higher kidney *Kim-1* mRNA levels, while *Ngal* was upregulated by IL-17A infusion (**Figure 2**). These data, evidencing subclinical kidney injury, suggest that the clinically significant kidney injury might eventually occur on longer follow-up.

**Figure 3:**
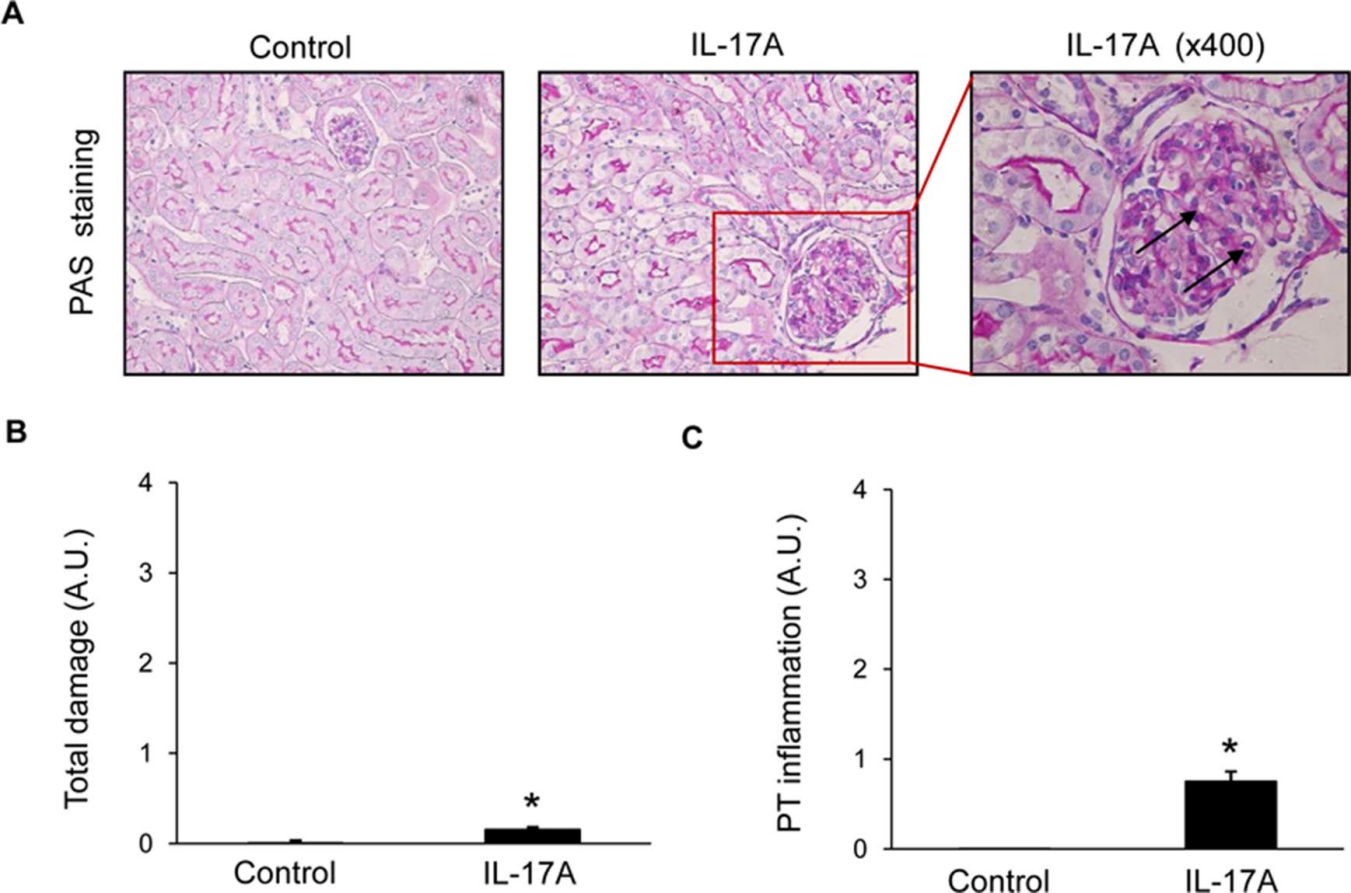
Kidney histopathology in mice with continuous systemic IL-17A infusion. Kidney sections were stained with PAS and evaluated by a pathologist in a blinded manner. Figure shows **(A)** representative images of light microscopy findings. There were no structural changes in IL-17A-infused mice compared with controls. Arrows represent inflammatory cell infiltration. Magnification ×200. Quantification of total renal damage **(B)** or peritubular inflammatory cell infiltration **(C)**. Figures show data as the mean ± S.E.M. of 6–8 mice per group. *p < 0.05 vs. control.

Histopathological evaluation of PAS-stained samples revealed no significant damage to parenchymal renal cells after 2 weeks of IL-17A infusion (**Figure 3**), no tubular injury (dilatation or atrophy) was found, the brush border was preserved in all mice, and there were no proteinaceous casts or evidence of interstitial fibrosis (**Figures 3A, B**). Interestingly, IL-17A-infused mice showed increased inflammatory cell infiltration (**Figure 3C**), including isolated leukocytes in scattered glomeruli (**marked by arrows in** **Figure 3A**).

**Figure 4:**
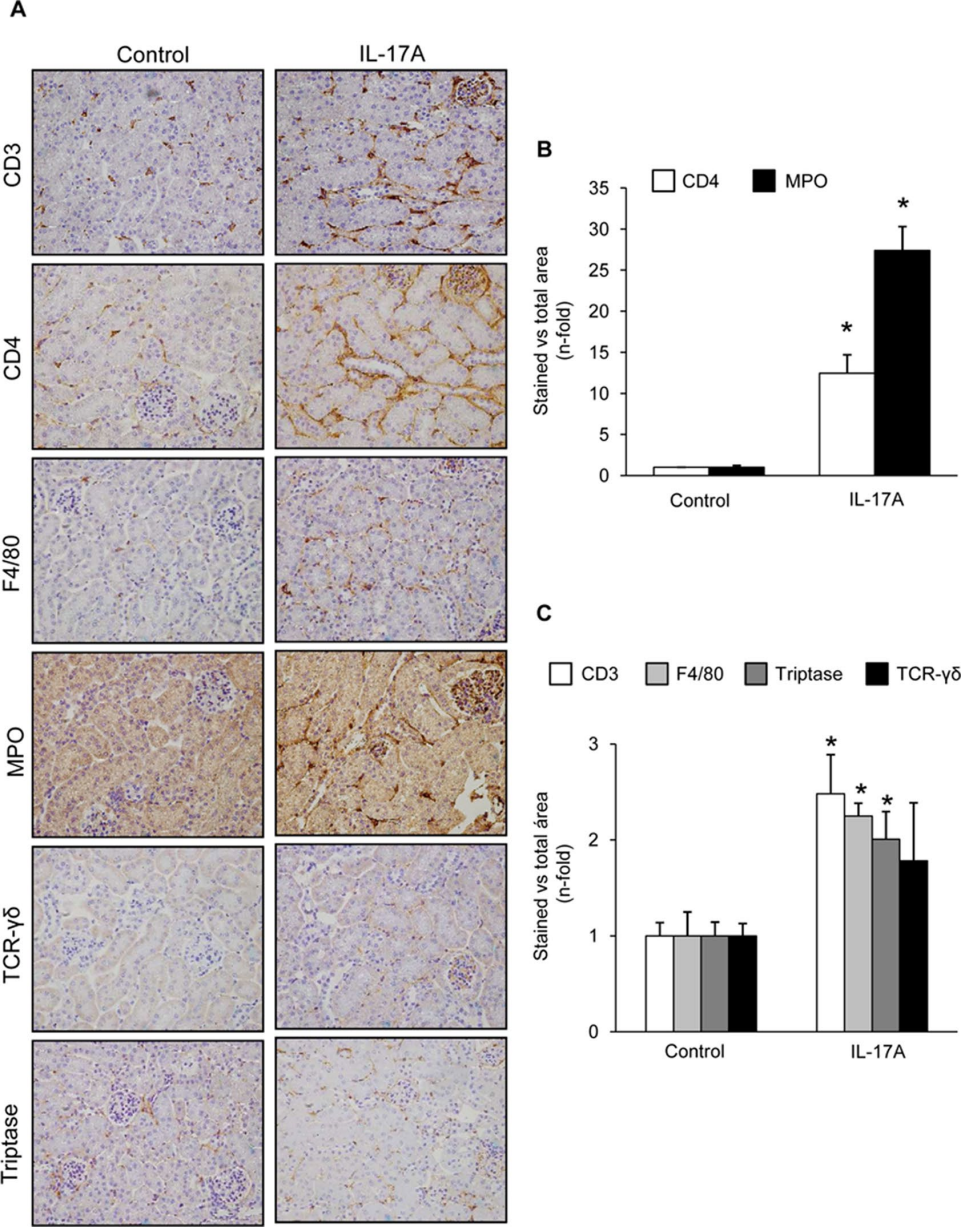
Systemic infusion of IL-17A in mice induces kidney inflammation. Inflammatory cell infiltration was characterized by immunohistochemistry using antibodies for monocyte/macrophages (F4/80), lymphocytes (CD3, CD4, and γδ), neutrophils (myeloperoxidase, MPO), and mast cells (tryptase). **(A)** Representative immunohistochemistry images of IL-17A-infused and control mice. **(B)** and **(C)**. Inflammatory cell staining was quantified as the percentage of total kidney area that was stained. Data are expressed as mean ± S.E.M. of 6–8 mice per group. *p < 0.05 vs. control.

**Figure 5:**
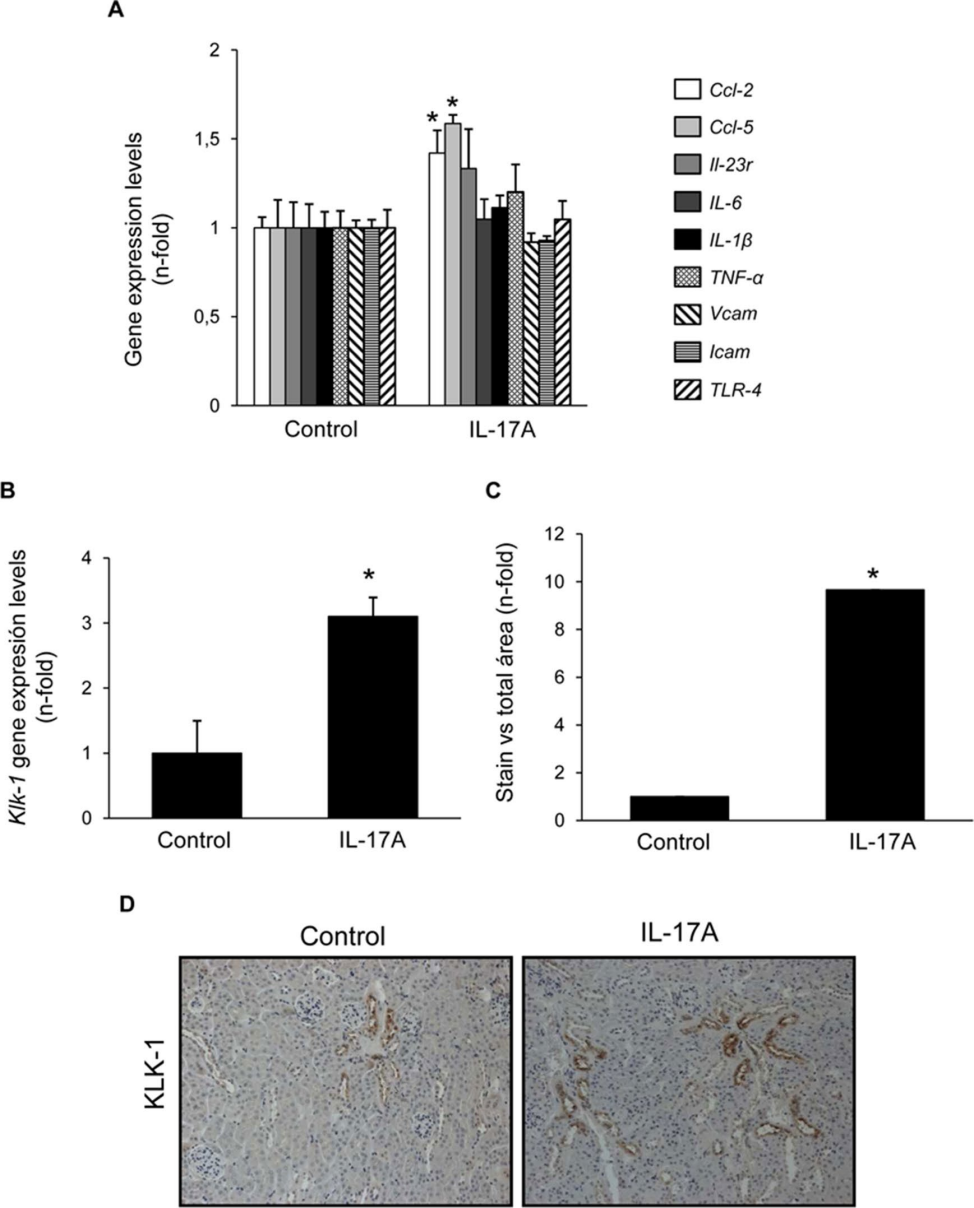
Systemic infusion of IL-17A in mice increases renal expression of proinflammatory factors. Evaluation of gene expression of proinflammatory factors **(A)** and kallikrein-1 **(B)** in the kidney of IL-17A-infused and control mice evaluated by real-time PCR gene. In paraffin-embedded sections, kallikrein-1 was evaluated by immunohistochemistry. **(C)**. Quantification of the staining. Figure **(D)** shows representative images of light microscopy showing positive tubular staining in IL-17A-infused mice. Data are expressed as mean ± S.E.M. of 6–8 mice per group. *p < 0.05 vs. control.

**Figure 6:**
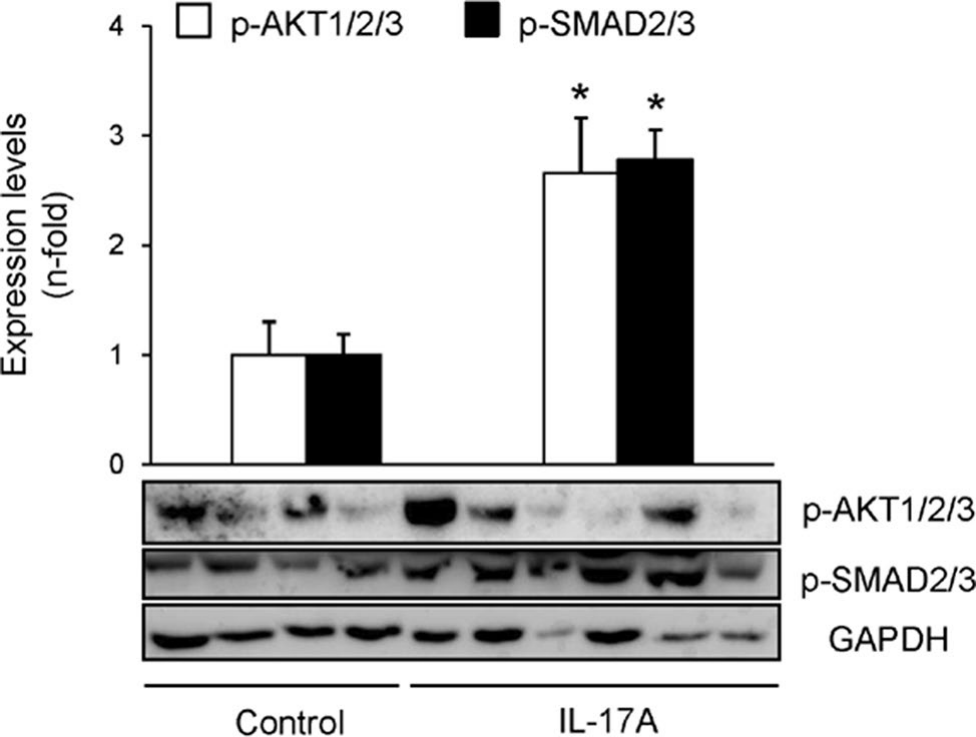
IL-17A administration in mice activates Smad and AKT signaling systems. Kidney protein levels were evaluated by Western blotting. Activation of intracellular pathways was assessed by the phosphorylation of Akt, Smad2/3. Glyceraldehyde 3-phosphate dehydrogenase or tubulin was used as a loading control. Figure shows several representative mice from each group and the quantification of the Western blot data. Data are expressed as mean ± S.E.M. of 6–8 mice per group. *p < 0.05 vs. control.

Cell infiltrates were further characterized by immunohistochemistry. T-lymphocytes, neutrophils, monocyte/macrophages, and mastocytes were observed in the kidneys of IL-17A-infused mice, whereas while few inflammatory cells were detected in controls (**Figures 4A–C**). To investigate the mechanisms involved in IL-17A-induced renal inflammatory cell recruitment, we evaluated the expression of genes encoding pro-inflammatory chemokines and cytokines. In IL-17A-infused mice, renal expression levels of *Ccl-2* (encoding MCP-1) and *Ccl-5* (encoding RANTES) were significantly increased when compared with the control group (**Figure 5A**). Some proinflammatory-related genes were slightly upregulated, as *IL-23r* mRNA,****whereas other genes were not changed, as the cytokines *il-6* and *il1-β* (**Figure 5A**). Interestingly, administration of IL-17A increased renal *kallikrein-1* gene and protein levels, observed by quantitative PCR, and immunohistochemistry (**Figures 5B–D**). Additionally, evidence of intracellular signaling activation (Smad and Akt pathways) was found in IL-17A-infused mice (**Figure 6**).

**Figure 7:**
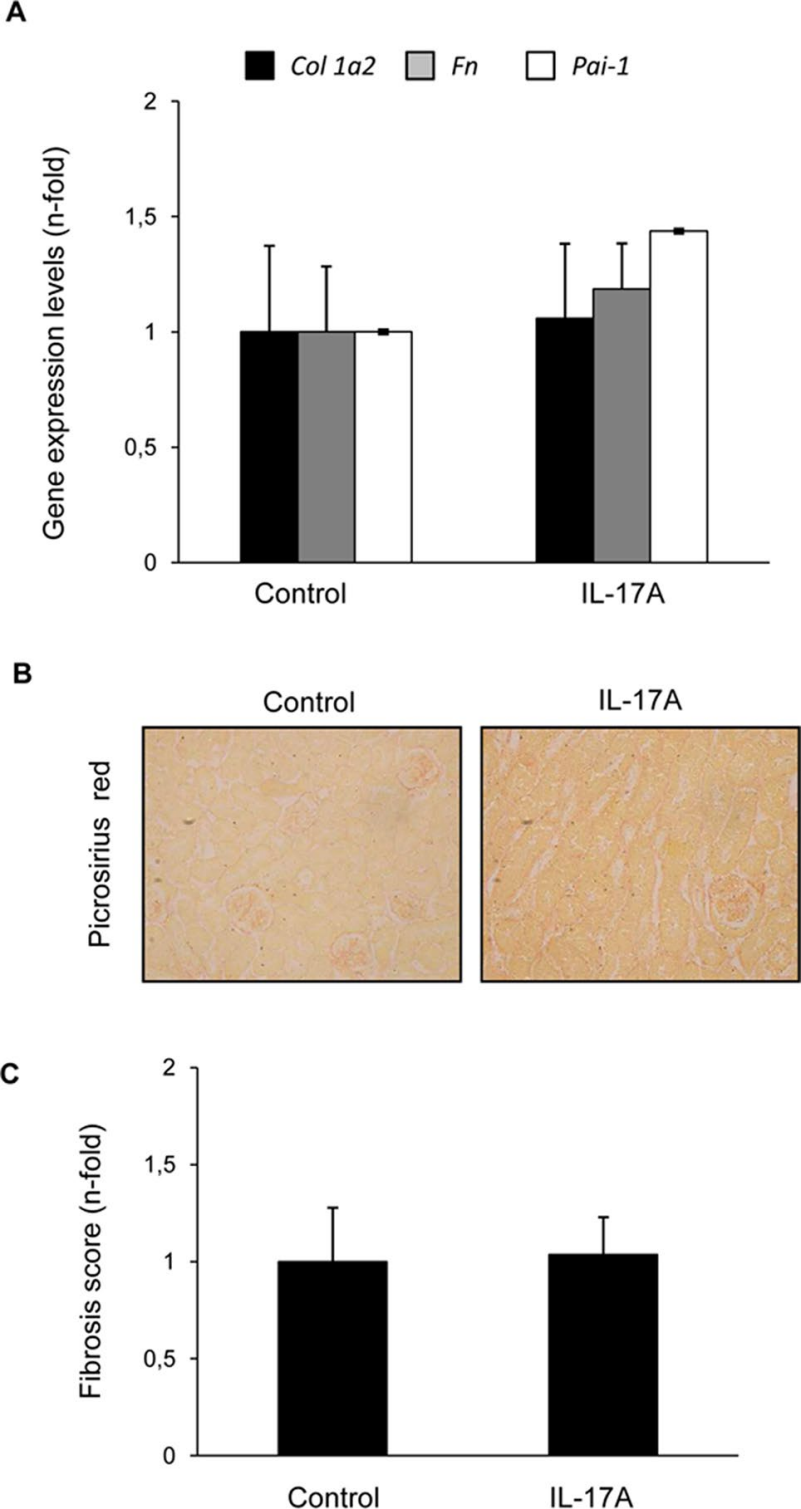
IL-17A administration in mice does not induce renal fibrosis within the time-course of the study. **(A)** In IL-17A-infused mice, the gene expression of profibrotic factors and ECM components was evaluated by real-time PCR. Collagen accumulation was evaluated in paraffin-embedded sections stained with Sirius Red. Figure **(B)** shows a representative mouse kidney and in **(C)** quantification. Data are expressed as mean ± S.E.M. of 6–8 mice per group. *p < 0.05 vs. control.

To further evaluate renal effects of systemic elevated IL-17A, gene expression encoding profibrotic factors or extracellular matrix components (ECM) was evaluated. There were no changes in the mRNA expression levels of the ECM components *type I procollagen* or *fibronectin*, neither in profibrotic factors, such as *PAI-1* (**Figure 7A**). This finding was consistent with the absence of kidney collagen accumulation, as assessed by Picrosirius red staining (**Figures 7B, C**).

### Treatment With an Anti-IL-17A Neutralizing Antibody Markedly Reduced Renal Inflammation in AngII-Infused Mice

**Figure 8:**
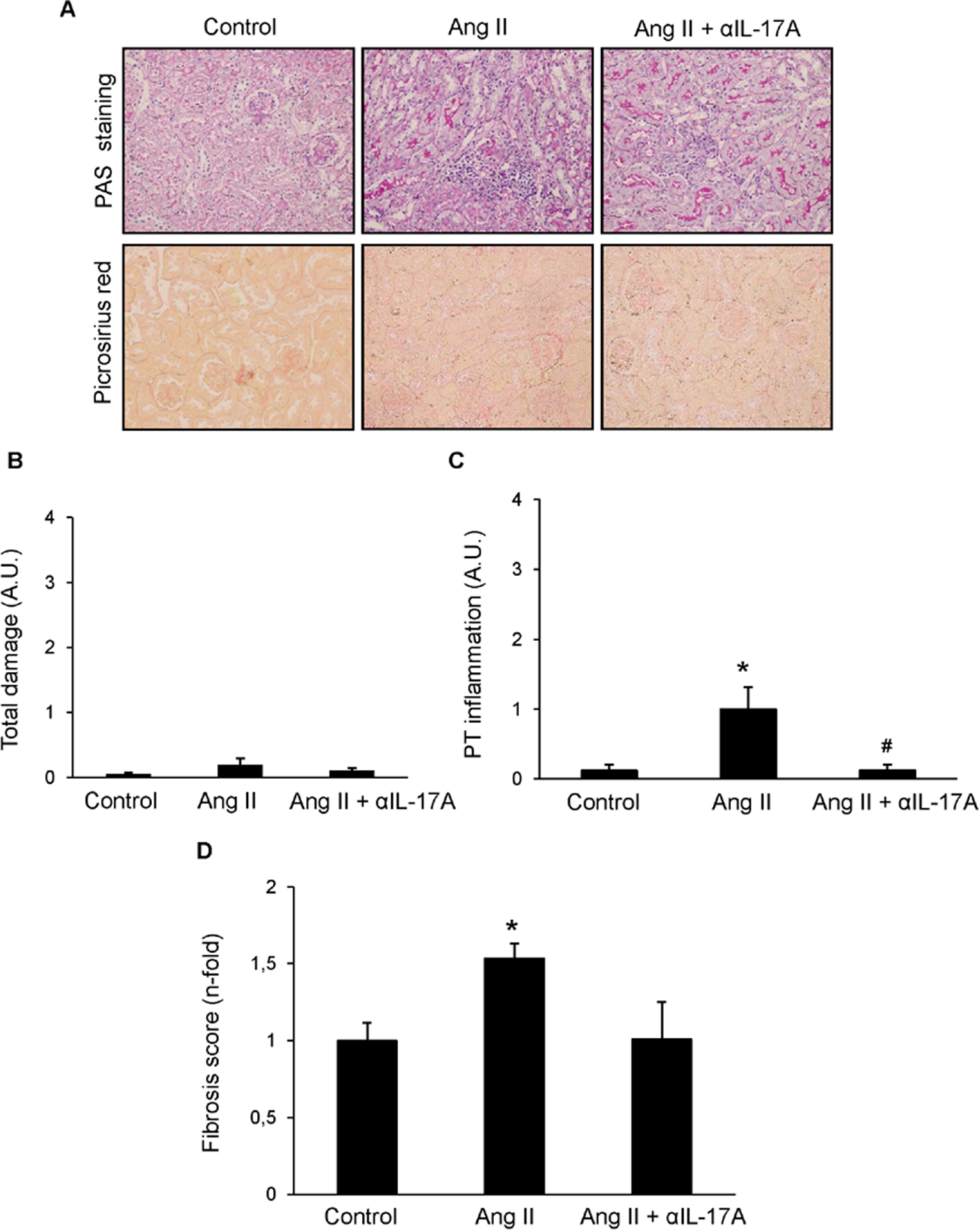
Effect of IL-17A neutralization on renal morphology and fibrosis in the model of angiotensin II infusion in mice. Mice were infused with 1,000 ng/kg/min angiotensin II (Ang II) for 14 days, treated or not with a neutralizing antibody against IL-17A (100 µg/mouse i.p. every 4 days), and compared with control mice. **(A)** Kidney sections were stained with PAS and picrosiruis red. Figures show representative images of light microscopy. Magnification ×200. The quantification of renal lesion **(B)**, inflammatory infiltration **(C)**, and collagen **(D)** is also shown. Data are expressed as mean ± S.E.M. of 6–8 mice per group. *p < 0.05 vs. Control. ^#^p < 0.05 vs. AngII-infused mice.

**Figure 9:**
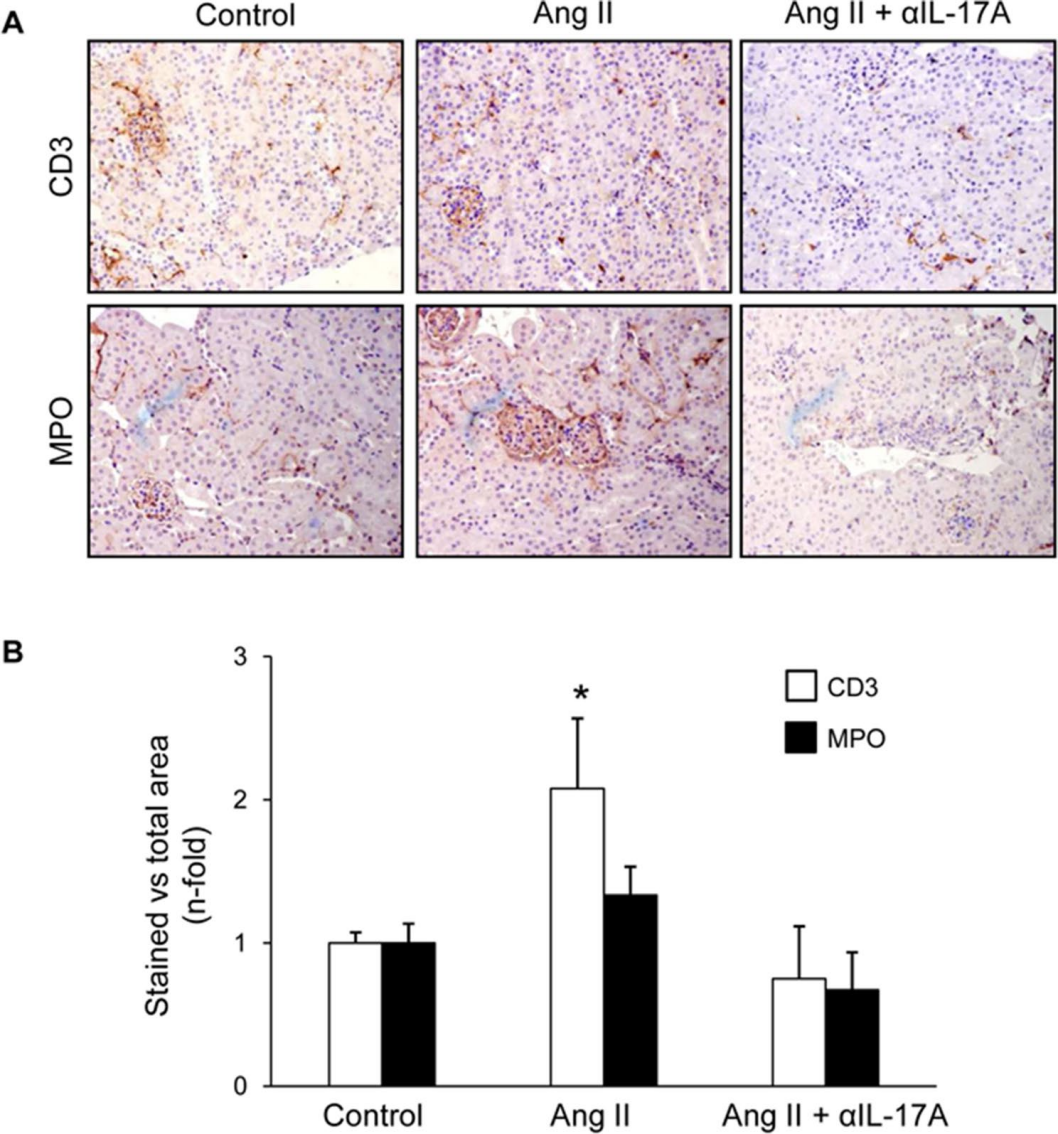
IL-17A neutralization diminished kidney inflammatory cell infiltration to Angiotensin II infusion in mice. **(A)** Representative images evaluating the presence of CD3^+^ cells and neutrophils in control, AngII-infused and AngII-infused + Anti-IL-17A-treated mice and in **(B)** data quantification. Results are expressed as mean ± S.E.M. of 6–8 mice per group. *p < 0.05 vs. Control. #p < 0.05 vs. AngII-infused mice.

**Figure 10:**
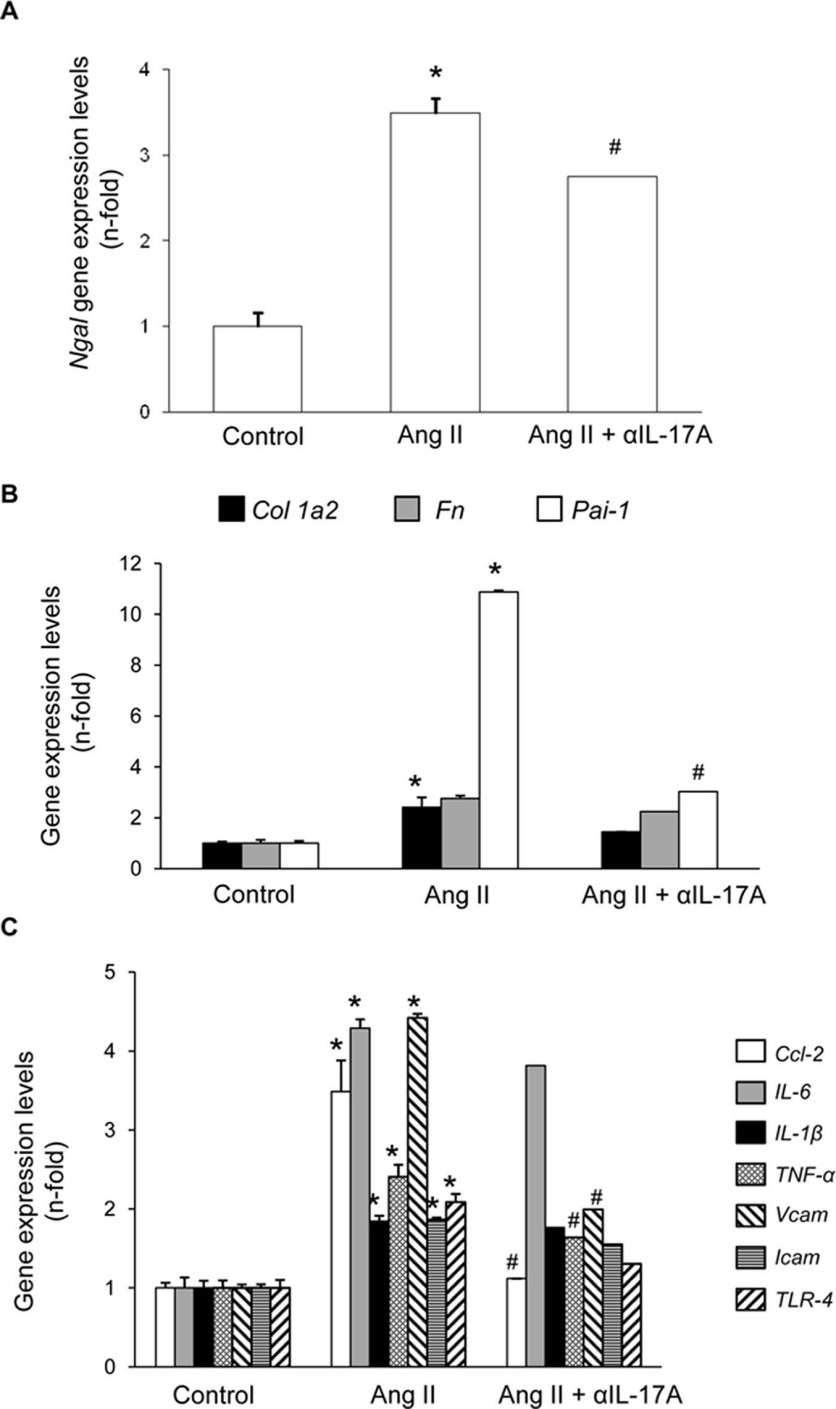
Effect of IL-17A neutralization on renal gene expression in Angiotensin II-infused mice. Figures show renal gene expresión levels of N-gal **(A)**, Collagen-1, Fibronectin, PAI-1 **(B)**, Ccl-2, Ccl-5, Il-23r, IL-6, IL-1β, TNF-α, Vcam, Icam and TLR-4 **(C)**. Renal mRNA expression was evaluated by real-time PCR. Figures show the mean ± S.E.M. of 6–8 mice per group. *p < 0.05 vs. Control. #p < 0.05 vs. AngII-infused mice.

Next, we tested whether IL-17A blockade with a neutralizing antibody modulated renal inflammation *in vivo* in the well-characterized model of sustained renal inflammation triggered by systemic AngII infusion in mice (Alique et al., 2014).,First, the histopathological injury was evaluated by PAS and Picrosirius red staining (**Figure 8**). In AngII-infused mice, no relevant renal damage was observed (**Figure 8B**) but significantly increased inflammatory cell infiltration (**Figure 8C**) and collagen deposition (**Figure 8D**) compared with controls was found. IL-17A neutralization decreased AngII-induced kidney inflammatory cell infiltration (**Figure 8C**) and diminished the presence of CD3^+^ T-lymphocytes and neutrophils (**Figures 9A, B**). Moreover, IL-17A blockade markedly decreased kidney gene expression induced by AngII, including lower expression of *Ngal* (**Figure 10 A**). Importantly, there was a significant downregulation of genes encoding pro-inflammatory fators (including the chemokine *ccl-2*, the adhesion molecule *vcam*, and the cytokine *tnf-α* and there is a tendency to decrease in *tlr-4*), supporting a key role of IL-17A in the regulation of inflammatory process in experimental renal damage (**Figure 10 C**). In contrast, IL-17A neutralization did not ameliorate AngII-induced collagen deposition, observed by Picrosirius red staining (**Figure 8D**). Accordingly, IL-17A blockade did not change gene expression levels of the ECM components fibronectin and collagen upregulated by AngII administration (**Figure 10 B**).

### Expression of IL-17A in Human Renal Biopsies From Patients With a Diagnosis of Hypertensive Nephroangiosclerosis

**Figure 11:**
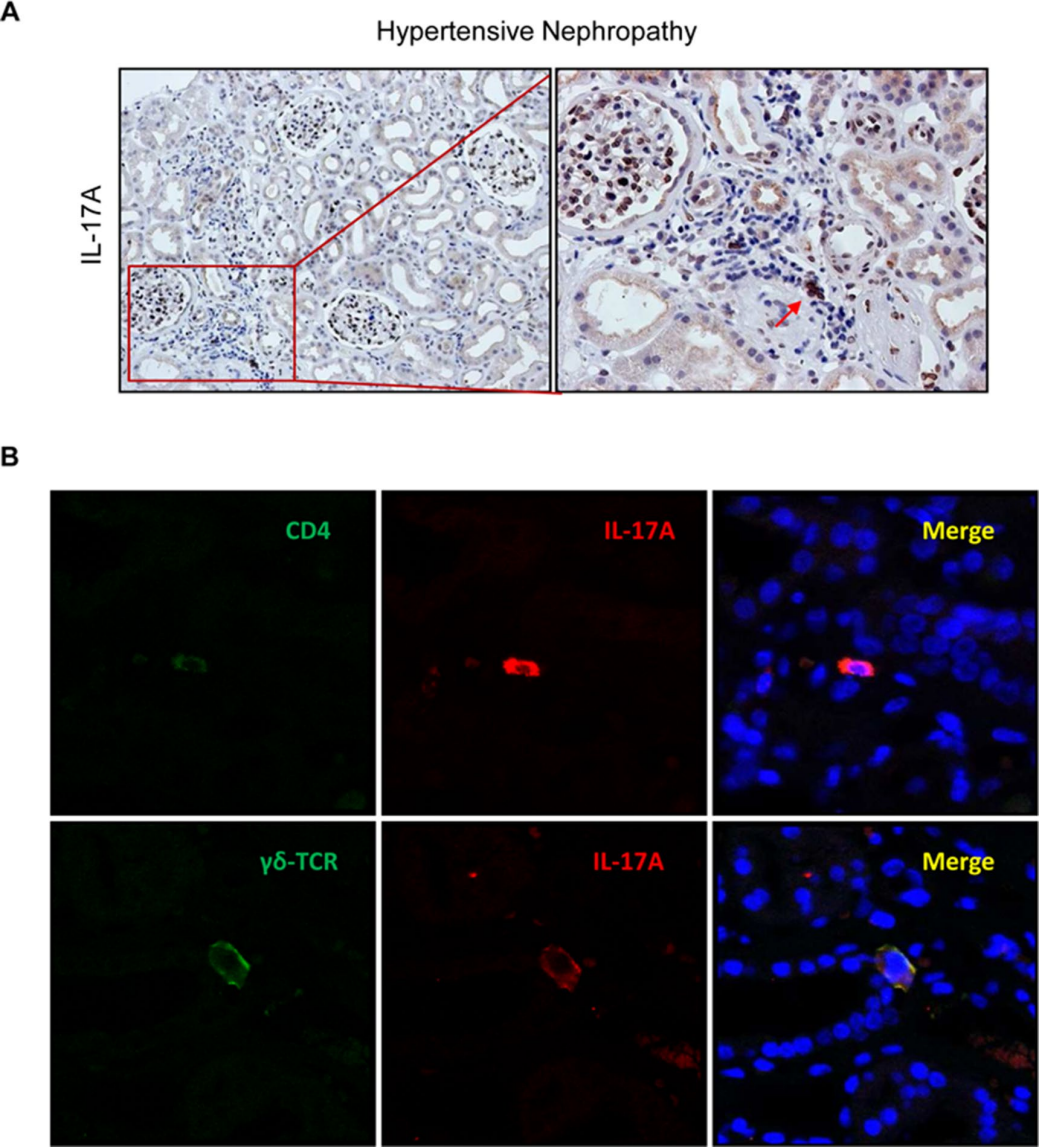
IL-17A-expressing cells in human hypertensive nephropathy. **(A)** IL-17A expressing cells detected by immunohistochemistry with anti-IL-17A antibody. Figure shows a representative image of human biopsies. **(B)** Characterization of IL-17A-expressing cells by double immunofluorescence for IL-17A, labeled by an Alexa 633 (red) secondary antibody and for CD4 (for CD4^+^/Th17 cells) or for γδT lymphocytes, both labeled with an Alexa 488 (green) secondary antibody. Nuclei were stained with 4,6-diamidino-2-phenylindole (DAPI; blue). The figure shows a confocal microscopy analysis of a representative patient out of 20 cases studied with similar results.

In renal biopsies of patients with a clinical and histopathological diagnosis of hypertensive nephroangiosclerosis, IL-17A positive cells were found localized mainly in areas of focal inflammatory cell infiltration (**Figure 11A**). Th17 cells were identified by confocal microscopy as CD4^+^/IL-17A^+^ cells (**Figure 11B**). Among other cell types producing IL-17A, IL-17A^+^/γδ T lymphocytes were also observed in diseased kidney (**Figure 11B**). Thus, IL-17A may be produced by different types of infiltrating leukocytes in patients with hypertensive kidney disease.

### Expression of Interleukin 17A in the Kidney of Hypertensive Animals

**Figure 12:**
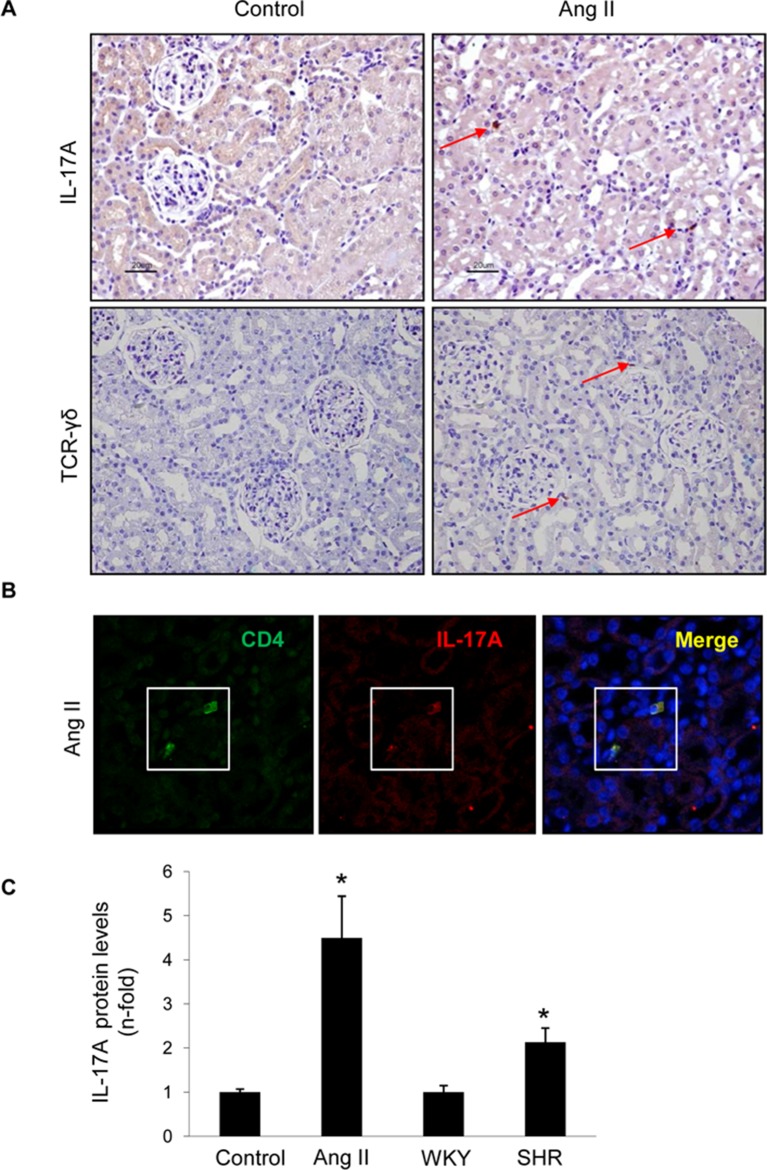
IL-17A-expressing cells in the kidney of rats with experimental hypertension. **(A)** The presence of IL-17A and γδ T lymphocytes was detected by immunohistochemistry. Figure shows a representative image. **(B)** Double immunofluorescence for IL-17A, labeled with an Alexa 633 (red) secondary antibody, and CD4 (for CD4^+^/Th17 cells) labeled with an Alexa 488 (green) secondary antibody. Nuclei were stained with 4,6-diamidino-2-phenylindole (DAPI; blue). **(C)** IL-17A protein levels were evaluated in total protein renal extracts by ELISA. The figure shows a confocal microscopy analysis of a representative rat of four studied with similar results. *p < 0.05 vs control.

The presence of IL-17A in hypertensive kidneys was also observed in two rat models: AngII infusion and SHR rats. In AngII-infused rat kidneys, positive IL-17A immunostaining was found, whereas no IL-17A signal was observed in control rat kidneys (**Figure 12A**). The presence of γδ T lymphocytes was also detected in AngII-infused rats (**Figure 12A**). Double immunostaining identified CD4^+^ lymphocytes as a source of IL-17A in hypertensive rat kidneys (**Figure 12B**). Moreover, ELISA of kidney homogenates demonstrated increased kidney IL-17A protein levels in both AngII-infused and SHR rats (**Figure 12C**).

## Discussion

Our experimental studies in a model of IL-17A systemic administration of low circulating concentration levels of this cytokine demonstrate the involvement of IL-17A in the recruitment of inflammatory cells into the kidney. Our data confirm and extend previous studies showing that IL-17A targeting decreased kidney inflammation in preclinical models of renal damage, suggesting that the blockade of IL-17A could be an anti-inflammatory treatment to prevent hypertension-induced renal inflammation.

Until now, there exist no studies demonstrating kidney parenchymal cells production of IL-17A. However, many cell types express IL-17A receptors. Several *in vitro* studies have described that IL-17A activates tubular epithelial cells, vascular smooth muscle cells, endothelial cells, and fibroblasts, to release a large array of proinflammatory mediators, including chemokines, such as MCP-1, RANTES, CXCL1, and CXCL8, which, in turn, may recruit T cells and macrophages leading to target organ injury (Van Kooten et al., 1998; Gaffen, 2009; Pietrowski et al., 2011; Zhang et al., 2013). IL-17A can also directly promote monocyte chemotaxis *in vivo* and *in vitro* (Sergejeva and Lindén, 2009; Shahrara et al., 2009), contributing to amplify the recruitment of immune cells to injured tissues. Accordingly, our *in vivo* results showed that systemic IL-17A administration significantly upregulated renal gene expression of MCP-1 and RANTES associated to the recruitment inflammatory cells to the kidney. Given the increased immune cell infiltration detected in the IL-17A-infused mice kidneys, overexpression of a large array of inflammatory mediators could be expected. However, mRNA levels of relevant proinflammatory cytokines, as *il-6* and *il1-β* were no changed by IL-17A systemic administration. Correspondingly, in the model of AngII-induced renal damage, those genes were not diminished by IL-17A neutralization treatment. In cultured cells, stimulation with IL-17A did not upregulate many proinflammatory genes by itself. However, IL-17A in combination with other inflammatory cytokines showed a synergist inflammatory effect (Erbel et al., 2009). Regarding cultured renal cells, IL-17A increases the production of MCP-1 in tubular epithelial cells (Van Kooten et al., 1998). MCP-1 is a key driver of inflammatory cell recruitment to the kidneys, mainly of monocyte/macrophages, and has been proposed as a biomarker of kidney damage. Indeed, MCP-1 blockade is nephroprotective in preclinical studies decreasing renal inflammation and is undergoing clinical trials for CKD (Tesch, 2008; Haller et al., 2016). Our *in vivo* data suggest that MCP-1 is a key target of IL-17A-mediated renal inflammation, as demonstrated by the significant upregulation at gene levels in IL-17A infused mice and by the inhibitory effect of IL-17A blockade in AngII-infused mice. These data suggest that MCP-1 could be a key downstream mediator of IL-17A renal actions and support the concept of IL-17A as a proinflammatory cytokine involved in renal inflammation.

Many studies mainly developed in vascular cells have investigated the molecular pathways activated by IL-17A. Most of them have focused on the inflammatory responses to IL-17A and have shown increased reactive oxygen species (ROS) production, modulation of nitric oxide levels, and activation of protein kinases, including RhoA/Rho-kinase and the MAPK cascade (Pietrowski et al., 2011; Xing, 2013; Zhang et al., 2013; Karbach et al., 2014; Wu et al., 2016). In this regard, IL-17A signaling has been extensively studied in preclinical atherosclerosis. However, both protective (Erbel et al., 2009; Smith et al., 2010) and deleterious effects (Akhavanpoor et al., 2017) have been described, illustrating the complexity of the system and potential contributions of the stage of the disease and the local microenvironment. Now, we have extended these mechanisms to the regulation of Smad and Akt pathways. All these data support the role of IL-17A in the regulation of inflammatory process in pathological conditions, including renal diseases and hypertension.

Experimental studies have demonstrated the involvement of T cells and T-cell-derived cytokines in the pathogenesis of hypertension (McMaster et al., 2015). Indeed, IL-17A-expressing cells were observed in target tissues of hypertension-induced damage, including the cardiovascular system and the kidneys (Madhur et al., 2010; McMaster et al., 2015; Saleh et al., 2016), as observed here in experimental hypertension in rats. In mice infused with AngII, T cell infiltrates were described in the adventitia and periadvential fat of the aorta associated with positive IL-17A immunostaining (Madhur et al., 2010). Kidney and aorta cells producing IL-17A were identified as Th17 and γδ T lymphocytes (Saleh et al., 2016). Interestingly, in murine AngII-induced hypertrophic hearts, γδ T lymphocytes are the main sources of IL-17A (Li et al., 2014). IL-17A production by γδ T cells has been involved in antifungal immunity and in the onset of autoimmune disease (Fenoglio et al., 2009; Hirata et al., 2011). Human IL-17A-producing γδ T cells are generated in the periphery and can be recruited to and accumulate in inflamed tissues, contributing to sustain inflammation (Shiromizu and Jancic, 2018). Importantly, our analysis of human kidney biopsies of hypertensive patients with nephroangioesclerosis showed positive IL-17A immunostaining, mainly in areas of interstitial inflammatory cell infiltration, identified as Th17 lymphocytes (CD4^+^/IL-17A^+^) and γδ T lymphocytes. The novel finding that γδ T cells express IL-17A in the kidney of hypertensive patients with nephroangiosclerosis remark the importance of additional studies evaluating whether specific pharmacological inhibition of γδ T cells could be used to treat hypertensive end-organ damage.

In this regard, blockade of the Th17/IL-17A axis has been suggested as a novel treatment for inflammatory kidney diseases. Beneficial effects of IL-17A blockade were described in preclinical immune- and nonimmune-mediated renal damage (Xue et al., 2011; Rodrigues-Díez et al., 2013; Peng et al., 2015; Ramani et al., 2016), as observed here by the anti-inflammatory effects observed in the model of AngII infusion. IL-17A blockers are tested for chronic inflammatory diseases, such as Crohn disease (NCT00936585), spondyloarthritis (NCT03358134), and psoriasis (NCT03403036). The presence of IL-17A-expressing cells in the kidneys of patients with hypertensive nephroesclerosis supports the clinical translation of our experimental findings and suggests that IL-17A blockade could be considered as a potential approach to prevent hypertension-induced kidney inflammation. In a recent study, we have found that therapeutic treatment with an anti-IL-17A neutralizing antibodies, starting after nephropathy, had already developed, improved kidney dysfunction and decreased kidney NF-κB activation, proinflammatory factors and inflammatory cell infiltration in experimental diabetic nephropathy in BTBR ob/ob (leptin deficiency mutation) mice (Lavoz et al., 2019), a model that uniformly develops human diabetic nephropathy features (Hudkins et al., 2010). The importance of inflammatory cytokines in the development of end-stage renal disease in diabetes has been recently demonstrated (Niewczas et al., 2019). Anti-inflammatory strategies are currently being explored in human clinical trials for diabetic kidney disease. Since hypertension frequently accompanies and accelerates CKD progression in other nephropathies, including the most common cause of end-stage renal disease, diabetic nephropathy, the clinical translation of our findings may not be limited to hypertensive kidney disease. However, only eventual clinical trials may answer the question of human translation.

Some evidences suggest that IL-17A contributes to fibrosis in several organs, such as skin and liver, but kidney data are contradictory (Ramani and Biswas, 2019). Targeting IL-17A or its receptor by gene deletion or pharmacological approaches in different experimental models of renal damage showed decreased or exacerbated fibrosis (Mehrotra 2017; Sun et al., 2018; Ramani et al., 2018). In our present study, neither ECM gene upregulation nor collagen accumulation was found in the kidneys of IL-17A-infused mice. In addition, IL-17A neutralization treatment did not ameliorate AngII-induced renal fibrosis. IL-17A increases ECM synthesis in several culture cells, including skin fibroblasts, but this cytokine could also contribute to ECM degradation by regulating metalloproteinases (Ramani and Biswas, 2019). In this regard, unilateral ureteral obstruction in IL-17 receptor knockout mice showed exacerbated renal fibrosis, increased renal kallikrein-1, and lower neutrophil, but not macrophages, infiltration that were restored by bradykinin treatment (Ramani et al., 2018). These authors suggest that IL-17 renal protection against fibrosis is due to metalloproteinase-2 diminution *via* kallikrein–kinin system regulated process. Other findings support the direct relation of IL-17A and kallikrein-1 in the kidney (Ramani et al., 2016). Accordingly, we have found that systemic IL-17A administration increased renal kallikrein-1 gene and protein levels, associated to neutrophils infiltration in the kidney, but no fibrosis.

Another important finding of our study is that IL-17A increased blood pressure. The mechanisms involved in the regulation of blood pressure are complex. Previous studies have demonstrated that IL-17A can directly induce hypertension associated to endothelial dysfunction, as observed in transgenic mice overexpressing IL-17A in keratinocytes (Karbach et al., 2014), or by intraperitoneal administration of high doses of recombinant IL-17A (Nguyen et al., 2013). The dose chosen in our study is expected to provide IL-17A exposure in the range described in pre-hypertensive patients (Yao et al., 2015) and suggests that low circulating IL-17A levels could be involved in the generation of hypertension. In this regard, IL-17A knockout mice were protected from Ang-II-induced increase in blood pressure. Moreover, AngII-induced changes in endothelium-dependent vasodilatation, phenylephrine-induced contraction, and ROS production were also prevented in IL-17A deficient mice (Madhur et al., 2010). RhoA/Rho-kinase and NO regulation contribute to IL-17A-mediated hypertension (Nguyen et al., 2013). Recent studies indicate that TLR-4 participates in hypertension (Biancardi et al., 2017) and in the regulation of experimental renal inflammation (Lin et al., 2012; Gonzalez-Guerrero et al., 2017). In TLR4 knockout mice, AngII-dependent blood pressure increase was blunted, in association with a diminution in ROS production, renal macrophage infiltration, and MCP-1 expression (Pushpakumar et al., 2017). We have observed that blockade of IL-17A significantly diminished renal *tlr-4* mRNA upregulation in AngII-infused mice, suggesting interplay between IL-17A and TLR-4 beneficial effects. Cytokines, including TGF-β1, TNFα, IFNγ, IL-1β, and IL-17A, may regulate blood pressure through effects on endothelial dysfunction, salt and water balance, and sympathetic regulation (Wen and Crowley, 2018). Accordingly, IL-17A-KO mice infused with AngII had preserved diuresis and natriuresis responses to an acute saline challenge (Kamat et al., 2015). However, IL-17A modulates the expression and activity of sodium transporters along the nephron. IL-17A deficiency abolished the activation of distal tubule transporters, specifically the sodium-chloride cotransporter and the epithelial sodium channel, and decreased AngII-induced renal damage (Norlander et al., 2016). Our study shows that IL-17A increases blood pressure, and therefore, additional studies are needed to further address the relation between IL-17A, hypertension, and renal inflammation.

Our present study has other limitations. The systemic IL-17A infusion in C57BL/6 mice increased blood pressure and promoted the recruitment of inflammatory cells into the kidney, but, at the dose and time-points studied, this did not result in biochemical or histological evidence of kidney disease beyond inflammation. Whether higher doses or longer exposure to IL-17A results in kidney disease should be addressed in future studies. In this regard, there was some evidence of subclinical parenchymal kidney injury. Thus, IL-17A-infused mice presented increased kidney *Ngal* expression. NGAL is expressed in the normal renal tubular epithelium and is overexpressed in AKI and CKD patients (Satirapoj, 2018). NGAL has been described as an early marker of kidney disease, since it is increased in urine during AKI before serum creatinine level elevation (Mori and Nakao, 2007; Devarajan, 2008; Parikh and Devarajan, 2008), and it is also increased in populations at high risk of CKD before other evidence of CKD develops (Ramírez-Rubio et al., 2016).

In summary, the present data support a role of IL-17A in kidney inflammation that highlights a potential contribution to regulation of hypertension. Our results suggest that IL-17A blockade could eventually be explored as an anti-inflammatory approach to prevent hypertension-associated end-organ injury.

## Data Availability

All datasets generated for this study are included in the manuscript and the supplementary files.

## Ethics Statement

All the procedures on animals were performed according to the European Community and Instituto de Investigación Sanitaria Fundación Jiménez Díaz Animal Research Ethical Committee guidelines. Studies were performed in adult male C57BL/6 mice (9–12 weeks old, 20 g). All patients provided informed consent and this study was conducted according to the principles of the Declaration of Helsinki and approved by the Institutional Review Board. Only tissue remaining after a pathological diagnosis achieved was used for research.

## Author Contributions

All the authors have reviewed the manuscript and approved the final version. MO and RRR-D contributed to the design of the experiments, acquisition, analysis and interpretation of all data, and drafted the manuscript. RR-D and AG-R contributed to design of the experiments, analysis and interpretation of data, and drafted the manuscript. LS-S and JR-G have participated in the development of mouse models, histology, immunohistochemistry experiments, and analysis of data. PC-O has evaluated all histological samples in a blinded manner. AMR, AO, RS, SM, and CL contributed to the critical review of the manuscript and the financial support of the work. MR-O contributed to the design of the experiments, analysis and interpretation of the all data, draft of the manuscript, and financial support of the experiments.

## Funding

This work was supported by grants from the Instituto de Salud Carlos III (ISCIII) andFondos FEDER European Union (PI17/00119, Red de Investigación Renal REDINREN: RD16/0009/0007, and CIBER-CV CB16/11/00286), Sociedad Española de Nefrologia, “NOVELREN-CM: Enfermedad renal crónica: nuevas Estrategias para la prevención, Diagnóstico y tratamiento” B2017/BMD-3751; PAI 82140017 and FONDECYT 1160465 (Chile). MO was a Fundación Conchita Rabago fellow and RR-D is supported by Postdoctoral MICINN Program IJCI-2017-31399; Spain.

## Conflict of Interest Statement

The authors declare that the research was conducted in the absence of any commercial or financial relationships that could be construed as a potential conflict of interest.
